# Photoinduced Strain‐Assisted Synthesis of a Stiff‐Stilbene Polymer by Ring‐Opening Metathesis Polymerization

**DOI:** 10.1002/chem.202002418

**Published:** 2020-10-15

**Authors:** Baiju P. Krishnan, Lulu Xue, Xinhong Xiong, Jiaxi Cui

**Affiliations:** ^1^ INM-Leibniz Institute for New Materials Campus D2 2 Saarbrücken 66123 Germany; ^2^ Institute of Fundamental and Frontier Sciences University of Electronic Science and Technology of China Chengdu Sichuan China

**Keywords:** isomerism, photoswitches, ring-opening metathesis polymerization, strain

## Abstract

Developing a novel strategy to synthesize photoresponsive polymers is of significance owing to their potential applications. We report a photoinduced strain‐assisted synthesis of main‐chain stiff‐stilbene polymers by using ring‐opening metathesis polymerization (ROMP), activating a macrocyclic π‐bond connected to a stiff‐stilbene photoswitch through a linker. Since the linker acts as an external constraint, the photoisomerization to the *E*‐form leads to the stiff‐stilbene being strained and thus reactive to ROMP. The photoisomerization of *Z*‐form to *E*‐form was investigated using time‐dependent NMR studies and UV/Vis spectroscopy. The DFT calculation showed that the *E*‐form was less stable due to a lack of planarity. By the internal strain developed due to the linker constraint through photoisomerization, the *E*‐form underwent ROMP by a second generation Grubbs catalyst. In contrast, *Z*‐form did not undergo polymerization under similar conditions. The MALDI‐TOF spectrum of *E*‐form after polymerization showed the presence of oligomers of >5.2 kDa.

## Graphical abstract

**A polymer** with several photoresponsive units is reported, which is of a great significance owing to the excellent stimuli‐response behavior. A photoinduced strain‐assisted ROMP of a macrocyclic π‐bond connected to a stiff‐stilbene through a linker is described. As the linker acts as an external constraint, photoisomerization to the *E*‐form causes stiff‐stilbene to be strained and thus to be reactive toward ROMP, resulting in a polymer with multiple stiff‐stilbene units.

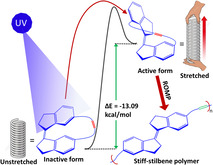

Polymers that respond to a light stimulus have drawn tremendous attention in recent years due to their immense potential in artificial muscles,^[1]^ photomechanical actuators,^[2]^ drug delivery systems,^[3]^ supramolecular systems,^[4]^ energy storage,^[5]^ switchable surfaces,^[6]^ and so on.^[7]^ Various types of molecular photoswitches, such as stilbene,^[8]^ azobenzene,^[9]^ spiropyran,^[10]^ diarylethene,[^2d^, ^11^] coumarin^[12]^ or fulgide,^[12]^ have been incorporated into the main or side chain of the polymer to achieve reversible photoresponsive polymers.^[13]^ Due to the light‐induced molecular changes, these molecular switches can cause a structural reconfiguration or change in the properties of polymer chains, which leads to fast, precise and remote control of various macroscopic properties, such as shape,^[2d]^ wettability,^[10b]^ optical properties,^[14]^ adhesion,^[15]^ solubility,^[16]^ conductivity,[^14^, ^17^] and so on. Compared to the side chain polymer, the main‐chain polymer made up of photoswitchable repeating units has improved thermal and mechanical properties that provide better photomechanical response.^[18]^ Despite significant advances in the development of photoresponsive polymers, the synthesis of main‐chain polymers necessitates complex synthesis of photoresponsive monomers, the use of toxic reagents and harsh conditions.^[19]^

Among various photoswitching molecules, 1‐(1‐indanyliden)indan, commonly known as stiff‐stilbene, has received much attention owing to 1) rigidity due to restricted rotation of phenyl ring, 2) remarkable thermal stability (a half‐life of ca. 10^9^ years at 300 K) due to high activation energy barrier between the two isomers (≈43 kcal mol^−1^), 3) superior photostability and chemical stability, 4) high quantum yield for the photoisomerization of either isomer (50 %) and 5) easily modifiable core structure.^[20]^ Boulatov and co‐workers employed a stiff‐stilbene force probe to determine the relationship between the restoring force in a stretched stiff‐stilbene macrocycle and the kinetics of dissociation of different functional groups within the macrocycle.[^20a^, ^20b^, ^21^] Apart from these force probe applications, they have been used for dynamic supramolecular polymerization,^[22]^ molecular machine,^[23]^ switchable catalyst,^[21]^ O_2_ sensor^[24]^ and stimuli‐responsive proton gate.^[25]^ Despite these encouraging applications, the design and function of the photoresponsive stiff‐stilbene polymers have not been achieved until now.^[26]^ Therefore, the development of new main‐chain stiff‐stilbene polymers is essential to gain more insight into this type of photomechanical system and to produce more advanced polymers whose physical properties and functions could be controlled by external stimuli. Herein, we report, for first time, photoinduced strain‐assisted ROMP synthesis of main‐chain stiff‐stilbene polymers by activating macrocyclic π bond connected to stiff‐stilbene photoswitch through photoisomerization.

Over the past years, ROMP has been an effective method for the synthesis of linear polymers from cyclic olefin of having a high angle or ring strains.^[27]^ For instance, in comparison studies of ROMP of various cyclic olefins (ring sizes 5–8), 6‐membered olefin (Cy6) was not subjected to ROMP due to the very low ring strain energies.^[28]^ The ring strain of a molecule can be manipulated if the ring structure is made up of a molecular photoswitch. For example, the ring‐opening reaction of cyclobutene connected to C6 and C6′ atoms in stiff‐stilbene through a linker are influenced by the force generated due to the increase of separation between C6 and C6′ atoms during photoisomerization, since stiff‐stilbene exhibits a tremendous changes in spatial extensions upon photoisomerization.^[20d]^ We envision that the stiff‐stilbene polymer can be synthesized by ROMP if the π bond is attached to the stiff‐stilbene through an aliphatic linker. In such a design, the π bond can be activated by photo‐triggered reversible spatial expansion^[29]^ due to the strain generated by the linker, which is an external constraint attached to C6 and C6” atoms in stiff‐stilbene when *Z*‐form isomerizes to *E*‐form (Figure 1).^[30]^

**Figure 1:**
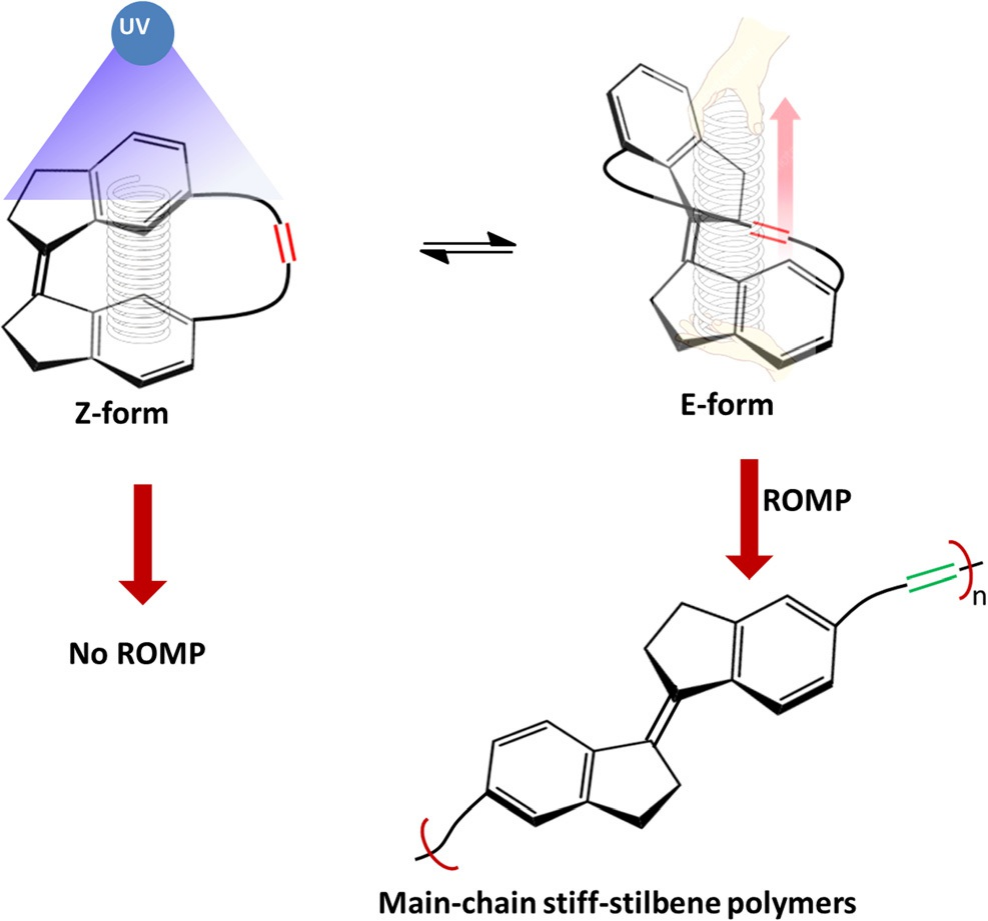
Schematic representation of photomechanical induced strain and subsequent ROMP for the synthesis of stiff‐stilbene polymer.

We synthesized the macrocycle **1** through a four‐step route with good yields (Figure 2 A and Scheme S1). Briefly, we have treated 2‐hexenoic acid (**2**) with 2nd generation Grubbs catalyst to yield *cis*‐4‐decenedioic acid (**3**). This diacid **3** further treatment with oxalyl chloride in the presence of *N*,*N*‐dimethylformamide (DMF) in dry dichloromethane (DCM) to yield corresponding acyl chloride which was further converted to 6‐hydroxy indinone diester (**4**) by treating 6‐hydroxy‐1‐indanone in dry DCM in the presence of triethylamine. The diester **4** was coupled each other using McMurry coupling with Zn dust/TiCl_4_ to yield the final macrocycle **1**.

**Figure 2:**
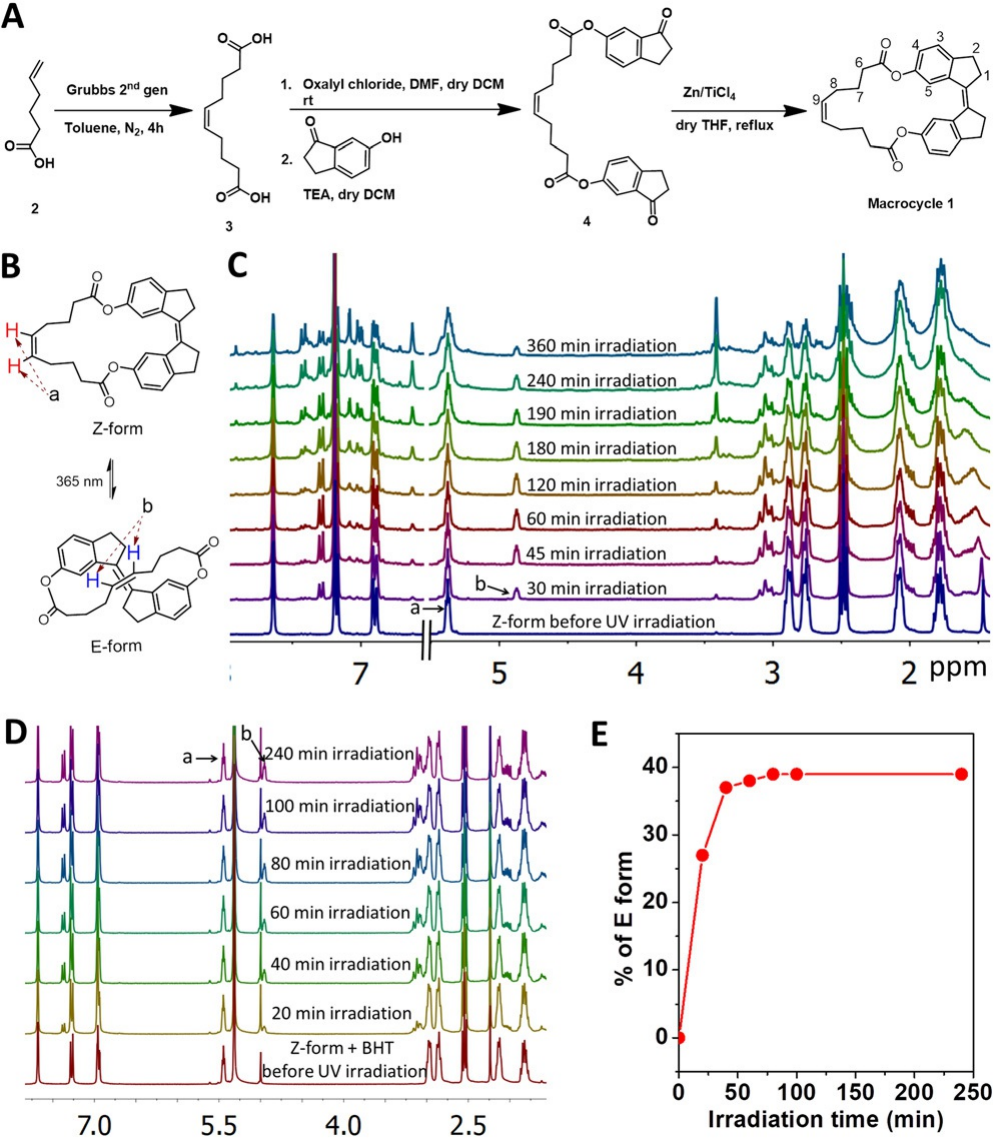
(A) Synthesis of macrocycle **1**. Carbons bearing hydrogen atoms are only numbered for better clarity; (B) photoisomerization of *Z*‐form to *E*‐form; (C,D) Time‐dependent ^1^H NMR spectra of *Z*‐form and mixture of *Z*‐form and BHT (inhibitor) during irradiation with 365 nm UV lamp, respectively; (E) Percentage of conversion of *E*‐form during the irradiation of the mixture of Z‐form and BHT.

The configuration of macrocycle **1** was studied by NMR spectroscopy. We expected a strong nuclear Overhauser effect (NOE) interaction between H‐1 and H‐5 (hydrogen atoms on carbons which are numbered in Figure 2 A) when the macrocycle is in the *E*‐form, but not in the *Z*‐form (Figure S1). The NOE spectrum shows that there was no interaction between H‐1 and H‐5, indicating that macrocycle **1** existed in the *Z*‐form, as shown in the previous reports.^[30]^ As expected, the *Z*‐form of macrocycle **1** was isomerized to the *E*‐form when its solution in chloroform‐*d*_3_ (0.014 m) was irradiated with UV light at a wavelength of 365 nm (Figure 2 B). Time‐dependent ^1^H NMR studies showed that the formation of a new olefin proton signal at 4.87 ppm (peak marked as b) is due to photoisomerization (Figure 2 C). Despite photoisomerization, prolonged UV irradiation also caused the initially formed *E*‐form to decompose, as can be seen from these NMR investigations. The ^1^H NMR showed that the olefin proton signal formed after photoisomerization was diminished with prolonged irradiation with the concomitant formation of many aromatic proton signals. This could be attributed to the random polymerization of macrocycle **1** double bonds. This decomposition was also observed in the other solvents (e.g., dichloromethane, Figure S2). Such undesirable UV‐initiated random polymerizations can easily be prevented by adding phenolic antioxidants that are known to react with free radicals. We added a butylated hydroxy‐toluene (BHT), a phenolic antioxidant (0.51 equivalents to macrocycle **1**) to the solution of macrocycle **1** in DCM‐d2 (0.014 m). This solution was irradiated with 365 nm UV lamp and analyzed by ^1^H NMR. A new olefin proton signal was formed at 4.85 ppm (peak labeled as b) due to the *E*‐form with a concomitant decrease in the signal at 5.40 ppm (peak labeled as a) due to the consumption of *Z*‐form as can be seen from the ^1^H NMR spectra (Figure 2 D). The kinetic transformation followed a hyperbolic relationship between the isomerization and the irradiation time (Figure 2 E). After a certain exposure time (100 minutes), the ratio of the Z‐form to the *E*‐form (0.38) remained unchanged, indicating a plateau stage, probably because the solution was able to reach equilibrium during this exposure time.

The UV/Vis spectroscopy gave additional evidence for the photoisomerization of macrocycle **1** in DCM solution (50 μm). Prior to irradiation, the absorption spectrum of macrocycle **1** showed two absorption peaks (*λ*_max_=338 nm and *λ*_max_=358 nm). Upon irradiation, these bands were blue‐shifted with an increase of molar extinction coefficient (*ϵ*), and the spectrum displayed a structured absorption with vibronic replicas with a maximum of 319 nm, 333 nm and 350 nm (Figure 3 A). The hypsochromic shift in absorption is due to the fact that the *E*‐form of the stilbene unit was not able to form a planar arrangement since the linker bound to C6 and C6” atoms serve as an external restriction. To compare the absorption spectrum of *E*‐form with an irradiated sample of *Z*‐form, pure *E*‐form was separated from the *EZ* mixture by column chromatography using 10 % ethyl acetate in petroleum ether as eluent. The chemical structure of *Z*‐form was confirmed by NOE measurement in which a strong interaction between H‐1 and H‐5 protons was found (Figure 3 B). The comparison of the absorption spectra of *E*‐form and irradiated *Z*‐form revealed that they were identical to each other. It is also known that the molar extinction coefficient (*ϵ*) of the *E*‐form is higher than that of the Z‐form.^[30]^ To compare *ϵ* values, both *Z*‐ and *E*‐form values were prepared at a concentration of 50 μm in DCM solvent. As expected, the separated *E*‐form had higher *ϵ* values up to 358 nm at all wavelengths.

**Figure 3:**
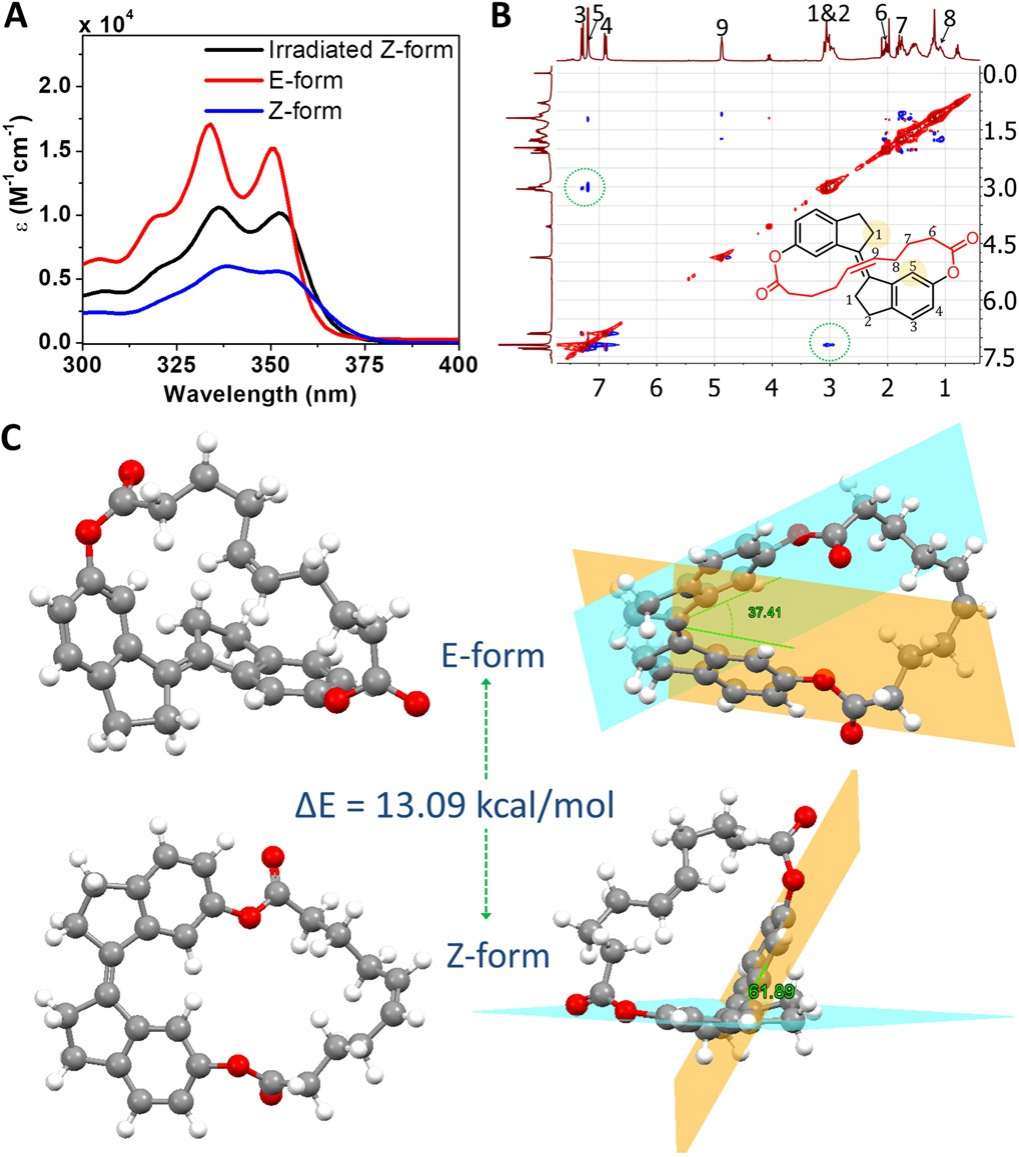
(A) Comparison of UV/Vis Spectra of *E*‐form, *Z*‐form and irradiated *Z*‐form with 365 nm UV lamp (30 min); (B) NOE spectra of pure *E*‐form, showing a strong interaction between H‐1 and H‐5 (only the carbons bearing H atoms are numbered); (C) Optimized structure of both *E* and *Z*‐forms with the angle between the planes of aromatic rings.

We also evaluated the stability of the *E*‐form in the *EZ* mixture obtained after irradiation. The irradiated sample was stored at room temperature under dark conditions for 12 hours. It is worth noting that the *E*‐form in the irradiated sample was stable under the dark condition as the signals from ^1^H NMR remained unaltered even after storage (Figure S3).

Density functional theory (DFT) calculation revealed that the *Z*‐form was more stable than the *E*‐form by a factor of 13.09 kcal mol^−1^ (Supporting Information). In contrast to unsubstituted stilbene, the *Z*‐form did not have planar geometry and the planes of the phenyl rings were tilted at an angle of 37.41°. In the *E*‐form, the angle between these planes was too large (61.89°), which caused the loss of conjugation as expected from UV/Vis spectroscopic studies (Figure 3 C). This large angle perturbation destabilized the *E*‐form. A number of macrocycles have shown that stretching disulfide bonds, sulfates, or esters accelerates the ring‐opening reaction with thiols, water, or reducing agents in their strained *E*‐form, making the stretched bonds easily cleavable.[^20b^, ^29^, ^31^] This is due to the reduction in the free activation energy with increasing strain on the macrocycles.^[31]^ We assume that the π bond bound to a stiff‐stilbene through a linker could be activated for ROMP through photoisomerization to synthesize the main‐chain stiff‐stilbene polymers.

To investigate the reactivity of *E*‐ and *Z*‐forms towards ROMP (Figure 4 A,B), the DCM‐*d*_2_ solution of *Z*‐form (0.014 m) was irradiated with 365 nm UV lamps for 60 minutes in the presence of BHT (0.51 equivalents to macrocycle **1**) to get the *EZ* mixture in which 37 % *E*‐form and 63 % *Z*‐form were present. The *EZ* mixture was then treated with 5 mol % 2nd generation Grubbs catalyst, and the kinetics of the reaction was studied by time‐dependent ^1^H NMR spectroscopy (Figure 4 C). It was observed that the reaction started immediately after the addition of the catalyst, which is evident from the appearance of new signals, especially those due to a new olefin at *δ* 5.45 (peak labeled as c in Figure 4 B). The intensities of these signals increased gradually over time, along with a concomitant reduction in the intensities of signals due to the *E*‐form (*δ* 4.87 due to olefin (peak labeled as b in Figure 4 B)). This trend continued until the reaction was 98 % complete in 159 minutes. A plot of percentage of the reaction against time showed that the reaction followed sigmoidal kinetics (Figure 4 D). Despite the ROMP in the E‐form, no significant change in the ^1^H NMR signals of *Z*‐form was observed. This could be indirectly attributed to the inability of the *Z*‐form of macrocycle **1** to open the ring for this polymerization by the catalyst because of its low strain energy.

**Figure 4:**
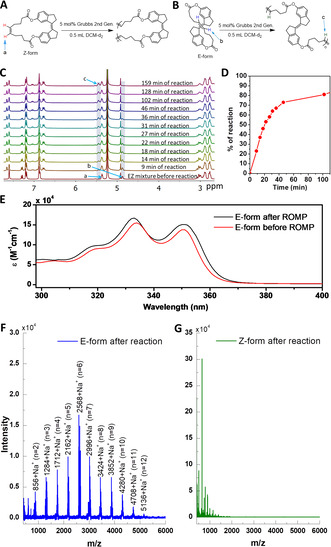
(A,B) Possible ROMP of *Z*‐ and *E*‐forms using 2nd generation Grubbs catalyst; (c) Time‐dependent ^1^H NMR spectra of irradiated sample of *Z*‐form after reaction with 5 mol % 2^nd^ generation Grubbs catalyst; (D) Plot of percentage of reaction of irradiated *Z*‐form vs. time; (E) Comparison of UV/Vis spectra of *E*‐form before and after ROMP; (F) MALDI‐TOF spectrum after reaction of *E*‐form with catalyst, showing the presence of oligomers up to 12‐mer; (G) MALDI‐TOF spectrum after reaction of *Z*‐form with catalyst.

There are two olefin bonds (stilbene and macrocyclic double bonds) present in the macrocycle **1**, and it is necessary to understand which bond actually opens by the catalyst. Though either of these bonds opens, the final structure of the polymer will be the same, but the newly formed double bonds may have different configurations (Supporting Information). Suppose stiff‐stilbene double‐bond is cleaved by the catalyst; the polymeric product must have mainly *Z*‐form of stiff‐stilbene units in its backbone, which can be easily investigated by UV/Vis spectroscopy. For example, cyclic ferrocenyl olefin (both *cis* and *trans* forms) underwent ROMP by 2nd generation Grubbs catalyst yielding polymers with only *cis* double bond configurations in their backbone.^[32]^ To prove this, we have separated *E*‐form from the *EZ* mixture. We also carried out ROMP of pure *E*‐ and *Z*‐form of macrocycle **1**. As observed in the *EZ* mixture, the pure *E*‐form also underwent ROMP, as can be seen from NMR spectra (Figure S4). The UV/Vis spectrum of *E*‐form after treatment with the catalyst was similar to that before the catalyst treatment, which indicates that the *E*‐configuration at stiff‐stilbene was unaltered even after polymerization (Figure 4 E). A slight red‐shift might be due to the increase in the conjugation by the ring‐opening reaction. This absorption spectrum was in good agreement with that of reported unmodified *trans*‐stiff‐stilbene.^[33]^ This reveals that only the macrocyclic double‐bond underwent ROMP by the catalyst, and the stiff‐stilbene double‐bond was still intact after polymerization.

The MALDI‐TOF mass spectrum of reaction mass after reaction of 160 minutes showed peaks with a spacing of 428 g mol^−1^ corresponding to oligomers from dimer to 12‐mers with an initial increase and then a gradual decrease in their intensities, indicating ROMP (Figure 4 F). The low degree of polymerization (DP) is due to the high catalyst loading (5 mol %) and DP can be increased by reducing the amount of catalyst. It is also noted that a very negligible amount of *Z*‐form was also formed during the reaction of *E*‐form with Grubbs catalyst, indicating negligible *E* to *Z* isomerization by catalyst (Figure S4). No significant change was observed in the reaction of the pure *Z*‐form with the 2nd generation Grubbs catalyst, except for the formation of new aromatic proton signals due to the isomerization reaction (Figure S4). Electrospray ionization mass spectrometry (ESI‐MS) and MALDI‐TOF spectra showed additional evidence to support this claim. In ESI‐MS and MALDI‐TOF spectra, most of the strong peaks, together with the monomeric peak, were below 1 kDa, indicating that even oligomers were not formed by the reaction of the Z‐form with the Grubbs catalyst (Figure 4 G and Figure S5).

In summary, we have developed a novel strategy to synthesize main‐chain stiff‐stilbene polymers using ROMP by inducing a strain to the macrocyclic π bond attached to a stiff‐stilbene through photoisomerization. The *Z*‐form of macrocycle **1** was photoisomerized with a maximum conversion of 38 % to the *E* form. The DFT calculation showed that the *E*‐form was more strained due to a lack of planarity, and therefore, the photoisomerization of the *Z*‐form could produce a strongly strained *E*‐form. Due to this high internal strain, *E*‐form in the *EZ* mixture underwent ROMP in the presence of a 2nd generation Grubbs catalyst. In contrast, the *Z*‐form did not undergo any polymerization. The MALDI‐TOF spectrum of the *E*‐form after treatment with the Grubbs catalyst indicated the formation of oligomers up to 12‐mer. This photoinduced strain‐assisted synthesis could be of great importance in the field of stimuli‐responsive polymer materials since these types of advanced polymers could have excellent photoresponsive properties due to the multiple stiff‐stilbene units present in their polymer backbone.

## Conflict of interest

The authors declare no conflict of interest.

## Supporting information

As a service to our authors and readers, this journal provides supporting information supplied by the authors. Such materials are peer reviewed and may be re‐organized for online delivery, but are not copy‐edited or typeset. Technical support issues arising from supporting information (other than missing files) should be addressed to the authors.

Supplementary
