# Supplementary material for: Photoinduced Strain‐Assisted Synthesis of a Stiff‐Stilbene Polymer by Ring‐Opening Metathesis Polymerization
Source: Chemistry. 2020 Oct 15;26(65):14828–32. doi: 10.1002/chem.202002418 (PMC7756494; doi:10.1002/chem.202002418)
Supplement: Supplementary file 1 — Supplementary [file CHEM-26-14828-s001.pdf]

Chemistry–A European Journal

Supporting Information

Photoinduced Strain-Assisted Synthesis of a Stiff-Stilbene Polymer by Ring-Opening Metathesis Polymerization

Baiju P. Krishnan,[a] Lulu Xue,[a] Xinhong Xiong,[a] and Jiaxi Cui*[a, b]

S. No.	Table of Contents	Page No.
1	Materials and methods	S2
2	Synthesis	S2
	2.1. Synthesis of compound **3**	S2
	2.2. Synthesis of compound **4**	S3
	2.3. Synthesis of macrocycle **1**	S3
3	NOE experiment: Evidence for the confirmation of Z-form	S4
4	Procedure for isomerization	S5
	(a) For NMR studies	S5
	(b) For UV studies	S6
	(c) For isolation of E-form	S6
5	Percentage of Z to E inter-conversion	S6
6	Stability of E-form in the EZ mixture	S7
7	ROMP of macrocycle **1**	S7
8	^{1}H NMR comparison of reactions of Z- and E-forms with Grubbs catalyst	S9
9	ESI-MS spectra of Z-form before and after the reaction	S10
10	Spectra	S11
11	References	S18

1. Materials and methods

Chemicals and solvents were purchased from Sigma-Aldrich and were used without further purification. The NMR spectra of the products were obtained with Bruker 300 MHz nuclear magnetic resonance equipment using $CDCl_3$ and CD_2Cl_2 as solvents. The column chromatography was carried out using 200-400 mesh silica gel. Elemental analyses were done on Elementar, vario MICRO cube elemental analyzer. UV/VIS Spectra were recorded with a Varian Cary 4000 UV/VIS spectrometer (Varian Inc. Palo Alto, USA). ESI-MS spectra were recorded using a QTos Ultima 3. The LED-UV monochromatic lamp LTR 365 (365 nm, 0.62 mW cm^{-2}) was used to irradiate the solutions. Density functional theory (DFT) calculations were performed using the Gaussian 09[1] program with the B3LYP hybrid functional[2] and basis set 6-311+G(d,p)[3] for the ground state geometry optimization. MALDI-TOF experiments were performed with rapifleX (Bruker) MALDI-ToF/ToF. For MALDI-TOF and UV-Vis experiments, all samples were made by passing the DCM solution of the samples through a celite column three times, then removing the solvent and drying under reduced pressure prior to the experiment.

2. Synthesis

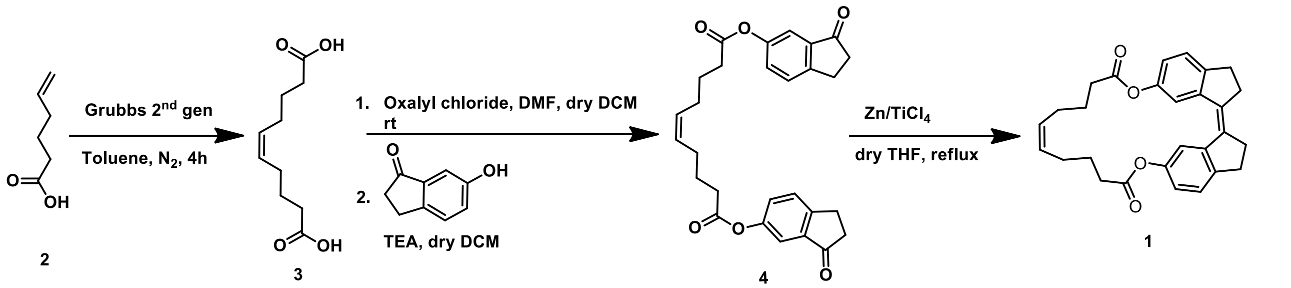

Scheme 1. Synthesis of macrocycle **1**

2.1. Synthesis of compound 3

A solution of **2** (1 g, 7.9 mmol) in dry toluene (6 mL) was purged with N_2 for 15 minutes. To the above solution, Grubbs catalyst, 2nd generation (67 mg, 79 µmol) was added and stirred under N_2 atmosphere. The reaction was monitored by TLC using an alkaline $KMnO_4$ stain. When the reaction was complete (4h), the mixture was directly loaded on a silica gel column and eluted with ethyl acetate-petroleum ether mixture (2:8 v/v) to yield the known diacid **3** (0.401 g, 51%) as a

white solid.[4] ^{1}H NMR (300 MHz, $CDCl_3$) δ 5.35–5.33 (m, 2H), 2.27 (t, J = 7.7 Hz, 4H), 2.01-1.95 (m, 4H), 1.64 (p, J = 7.4 Hz, 4H); ^{13}C NMR (75 MHz, $CDCl_3$) δ 180.08, 179.99, 130.22, 129.67, 33.30, 31.78, 26.37, 24.23. The characterization data are consistent with these previously reported.[4]

2.2. Synthesis of compound 4

To a solution of **3** (0.300 g, 1.5 mmol) in DCM (10 mL), oxalyl chloride (450 µL, 5.25 mmol) and dry DMF (1 µL, 0.013 µmol) were added and stirred for 2 hours. After completion of reaction, the solvent was removed under reduced pressure to get corresponding acid chloride and dissolved in dry DCM (5 mL). To a solution of 6-hydroxy-1-indanone (0.777 g, 5.24 mmol) in dry DCM (10 mL), triethylamine (831 µL, 6.00 mmol) was added and stirred for 10 minutes. Afterwards a DCM solution of acid chloride was added to the above solution at 0 °C and then stirred at room temperature for 12 hours. After completion of reaction, solvent was removed under reduced pressure and the crude solid thus obtained was chromatographed by using ethyl acetate-petroleum ether mixture (3:7 v/v) as eluent to yield compound **4** (0.550 g, 80%) as a white solid. ^{1}H NMR (300 MHz, $CDCl_3$) δ 7.41 (d, J = 7.4 Hz, 2 H), 7.36 (d, J = 2.5 Hz, 2 H), 7.22 (dd, J_1 = 8.3 Hz, J_2 = 2.3 Hz, 2 H), 5.44-5.40 (m, 2 H), 3.06 (t, J = 3.7 Hz, 4 H), 2.66 (t, J = 6.0 Hz, 4 H), 2.51 (t, J = 7.5 Hz, 4 H), 1.77 (p, J = 7.3 Hz 4 H). ^{13}C NMR (75 MHz, $CDCl_3$) δ 205.94, 170.05, 150.24, 150.17, 138.36, 130.28, 128.38, 127.49, 116.38, 36.86, 33.57, 31.82, 25.46, 24.60. Elemental analysis calculated for $C_{28}H_{28}O_6$, C = 73.03, H = 6.13; Found C = 73.17, H = 6.22.

2.3. Synthesis of macrocycle 1

To a stirred suspension of zinc powder (0.852 g, 13.08 mmol) in dry THF (ca. 50 ml), $TiCl_4$ (717 µL, 6.54 mmol) was added over 10 minutes at 0 °C. The resulting slurry was heated at reflux for 1.5 h. A THF solution of (ca. 50 ml) of compound **4** (0.500 g, 1.09 mmol) was added over a 2-3 h period using a dropping funnel to the refluxing reaction mixture. The reflux was continued for 30 minutes after the addition was complete. After cooling to room temperature, the reaction mixture was poured into a saturated aqueous solution of NH_4Cl, and the product was extracted with dichloromethane. The combined organic solutions were dried with anhydrous $MgSO_4$, the solvent was evaporated, and the crude product was chromatographed by using ethyl acetate-petroleum ether mixture (9:1 v/v) as eluent to yield macrocycle **1** as a white solid. ^{1}H NMR (300 MHz, $CDCl_3$) δ 7.63 (d, J = 2.1 Hz, 2 H), 7.18 (d, J = 8.4 Hz, 2 H), 6.89 (dd, J_1 = 8.4 Hz, J_2 = 1.8 Hz, 2

H), 5.39-5.35 (m, 2 H), 2.91-2.85 (m, 4 H), 2.78-2.73 (m, 4 H), 2.48 (t, J = 7.2 Hz, 4 H), 2.11-2.04 (m, 4 H), 1.82-1.73 (m, 4 H). ^{13}C NMR (75 MHz, $CDCl_3$) δ 172.44, 148.93, 145.59, 141.48, 135.49, 130.93, 125.48, 120.57, 116.13, 35.18, 32.30, 31.07, 30.04. Elemental analysis calculated for $C_{28}H_{28}O_4$, C = 78.48, H = 6.59; Found C = 78.52, H = 6.61.

3. NOE experiment: Evidence for the confirmation of Z-form

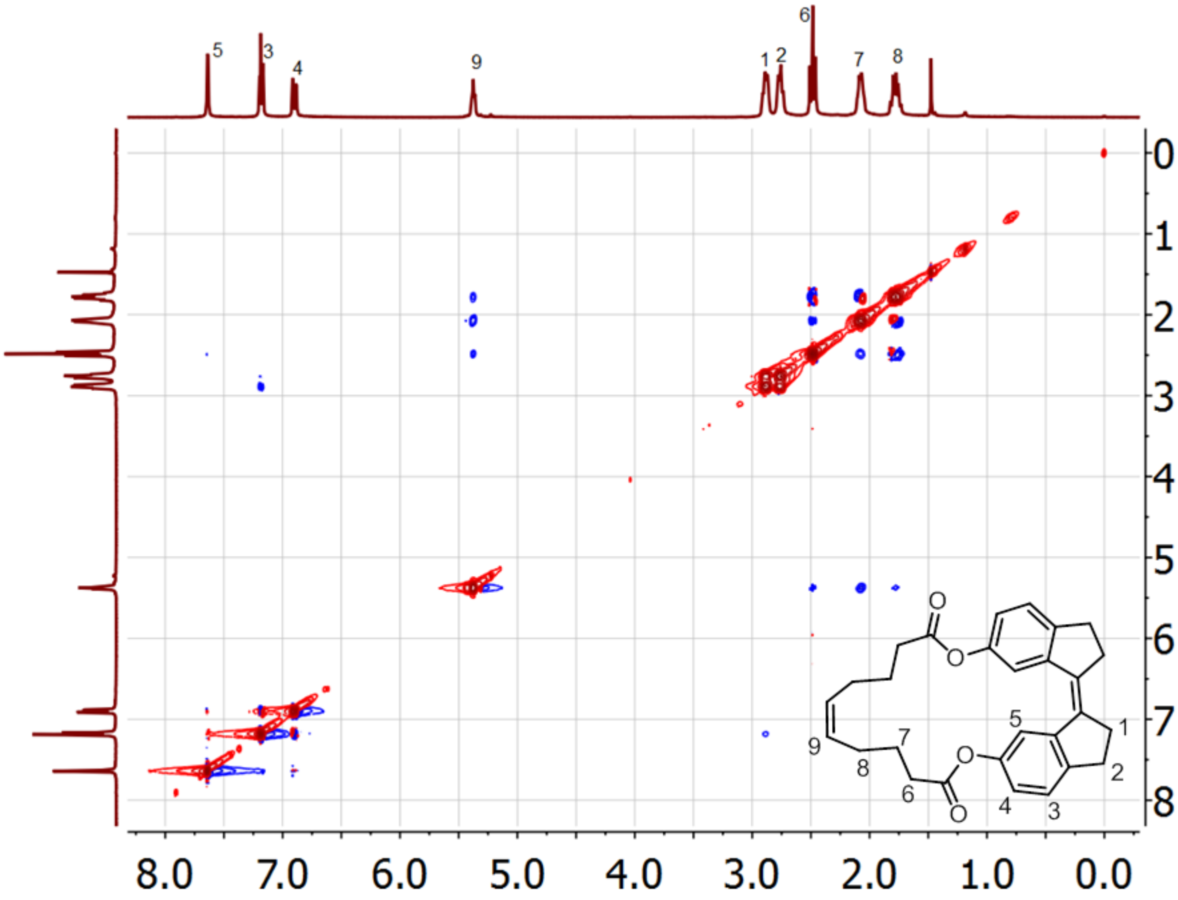

Figure S1. NOESY spectrum of Z-form showing no interaction between H-1 and H-5. Chemical structure of Z-form is shown in the down right of the figure. Carbons bearing hydrogen atoms are only numbered for better clarity.

4. Procedure for isomerization

(a) For NMR studies

Without BHT: The solution of macrocycle **1** (0.014) in $CDCl_3$ or CD_2Cl_2 was purged with N_2 for 5 minutes (Figure S2). After this purging, the solution was irradiated with UV light at a wavelength of 365 nm, and its 1H NMR was recorded at regular time intervals after syringing out to a NMR tube covered with aluminium foil.

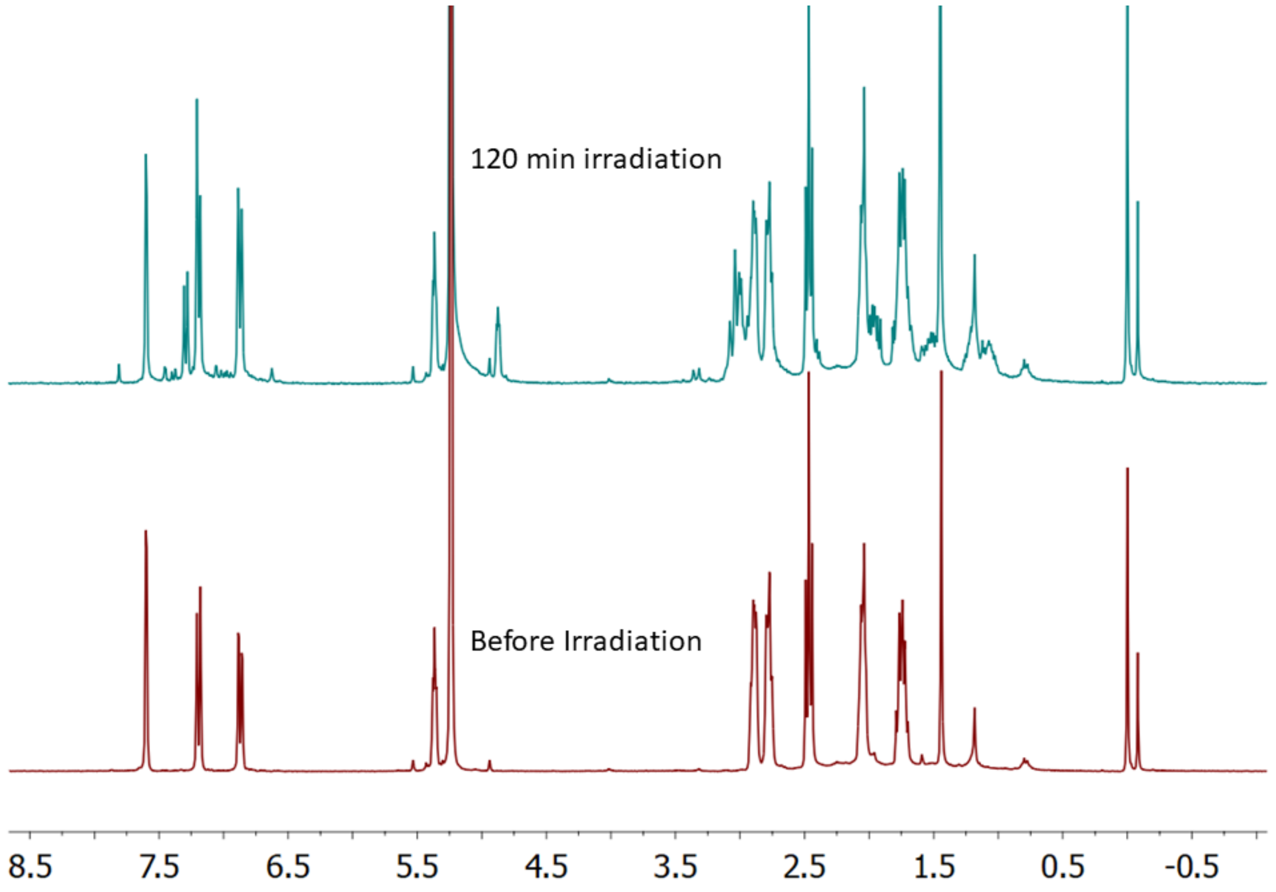

Figure S2. ^{1}H NMR spectral comparison of the dichloromethane-d2 solutions of macrocycle **1** before and after irradiation.

With BHT: A solution of macrocycle **1** (0.014 M) and BHT (0.007 M) was first prepared in CD_2Cl_2, followed by a 3 minute purging with N_2. This purged solution was then irradiated with 365 nm UV light, and its NMR was recorded at various time intervals by transferring the solution to a NMR tube covered with aluminium foil.

(b) For UV studies

A solution of macrocycle **1** (50 μM) and BHT (50 μM) was first prepared in dichloromethane, followed by a 3 minute rinse with N_2. This purged solution was then irradiated with 365 nm UV light and transferred to a 10 mm cuvette. The UV/Vis spectrum of this solution was recorded immediately.

(c) For isolation of E-form

The N_2-purged DCM solution of macrocycle **1** (50 μM) and BHT (50 μM) was irradiated with 365 nm UV light: After irradiation (60 minutes), the solvent was removed under reduced pressure and the crude product was chromatographed by using ethyl acetate-petroleum ether mixture (9:1 v/v) as eluent to yield E-form as a white solid. 20 mg of E-form was isolated from 100 mg starting material. This lower yield might be due to the loss of materials by column chromatographic separation or reversible E to Z conversion. During this process, all apparatus (column and round bottom flask) were covered with aluminum foil. ^{1}H NMR (300 MHz, CD_2Cl_2) δ 7.29 (d, J = 8.3 Hz, 2 H), 7.20 (d, J = 2.3 Hz, 2 H), 6.87 (dd, J_1 = 8.2 Hz, J_2 = 2.3 Hz, 2 H), 4.89-4.86 (m, 2 H), 3.11-2.90 (m, 8 H), 2.02-1.91 (m, 3 H), 1.82-1.67 (m, 5 H), 1,15-1.00 (m, 4 H). ^{13}C NMR (75 MHz, CD_2Cl_2) δ 172.49, 150.94, 145.75, 144.51, 135.19, 129.96, 126.17, 120.07, 117.80, 34.34, 33.33, 32.76, 31.38, 26.59.

5. Percentage of Z to E inter-conversion

The percentage of conversion was measured from the ^{1}H NMR integral of peaks 'a' and 'b' labeled in the manuscript. The equation for the calculation of the conversion is as follows:

$$\%\, of\ conversion = \frac{b}{a+b} X100$$

6. Stability of E-form in the EZ mixture

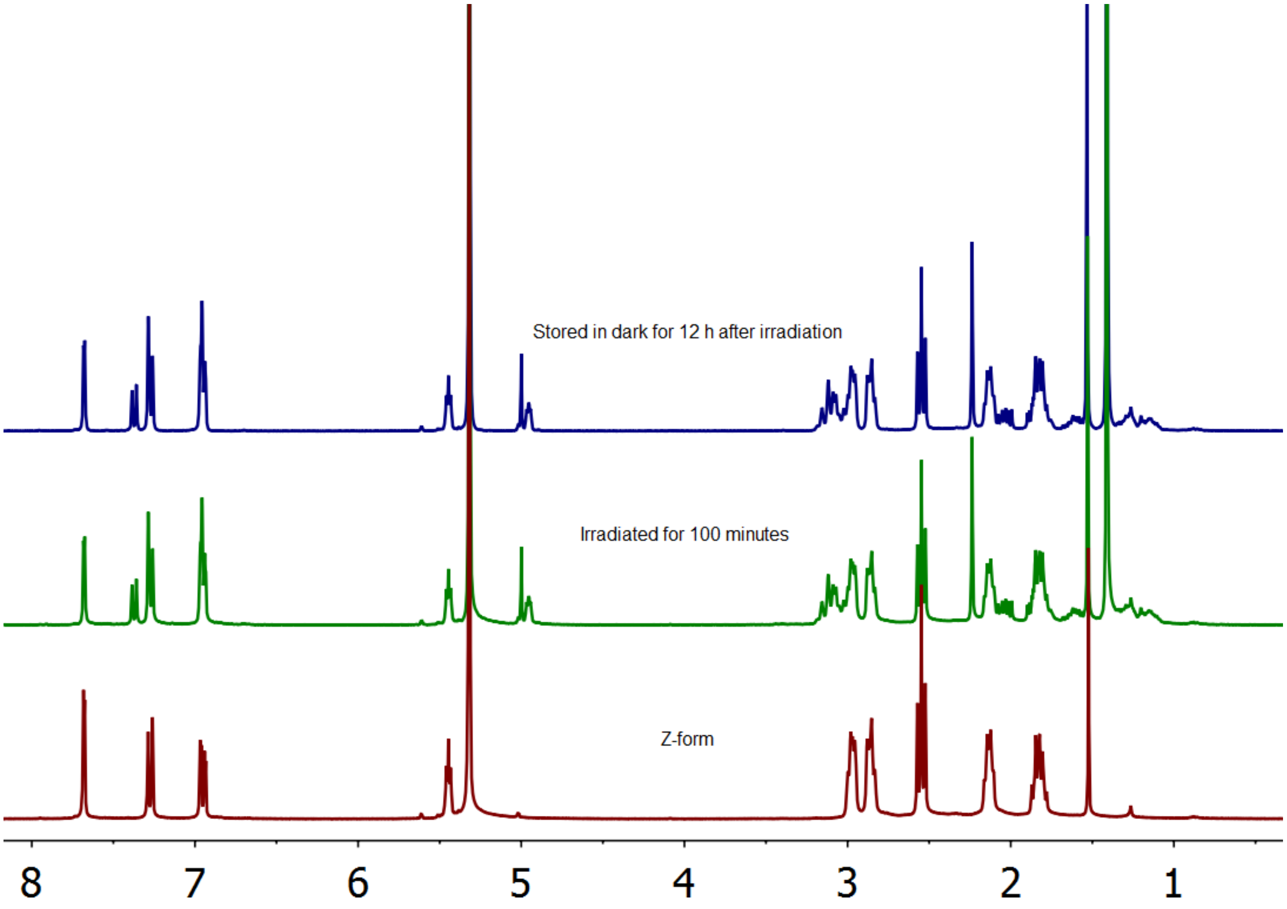

Figure S3. ^{1}H NMR comparison of Z-form, irradiated Z-form and stored sample after irradiation.

7. ROMP of macrocycle 1

A solution of macrocycle **1** (0.014 M) and BHT (0.007 M) was prepared in CD_2Cl_2, followed by purging with N_2 for 3 minutes. This purged solution was then irradiated with 365 nm UV light for 60 minutes. This mixture was transferred to a NMR tube, which was protected from light, and 5mol% catalyst was added to this NMR tube. The reaction kinetics was followed by recording its ^{1}H NMR at different time intervals during the progress of the reaction. The % of conversion was measured from ^{1}H NMR integral of peaks ‘c’ and ‘d’ labeled in the manuscript. The equation for the calculation of conversion is as follows:

$$\%\ of\ conversion = \frac{d}{c+d} X100$$

There are two double bonds present in macrocycle **1** and therefore there might be two possibilities for ROMP.

(a) ROMP by macrocyclic double bond:

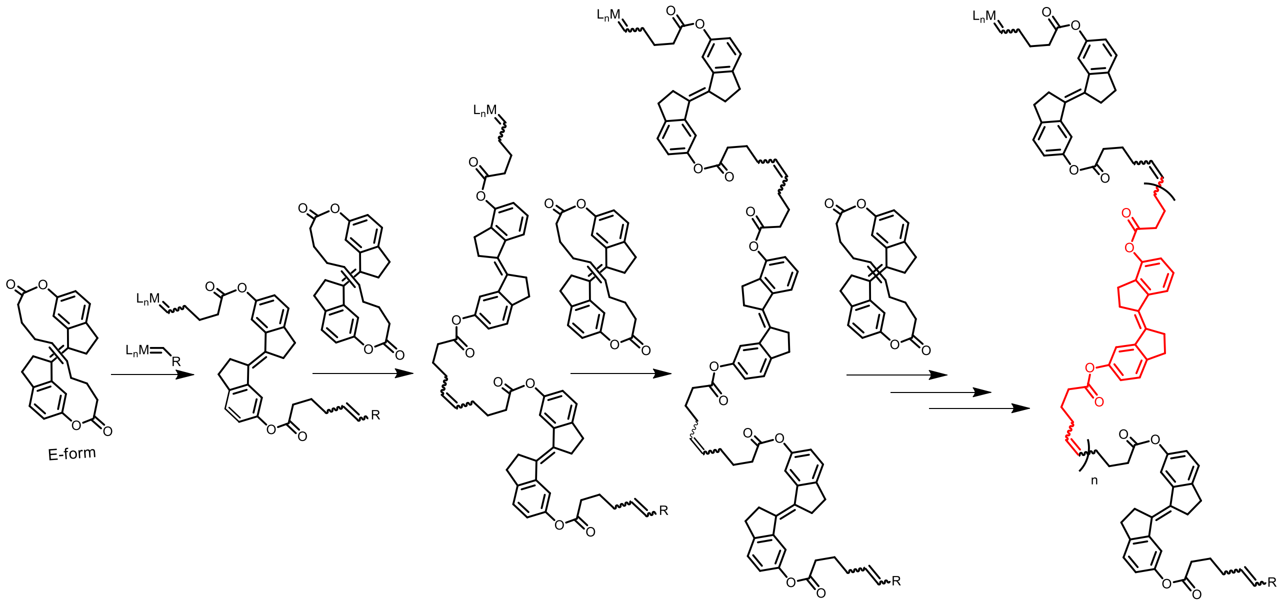

(b) ROMP by stiff-stilbene double bond:

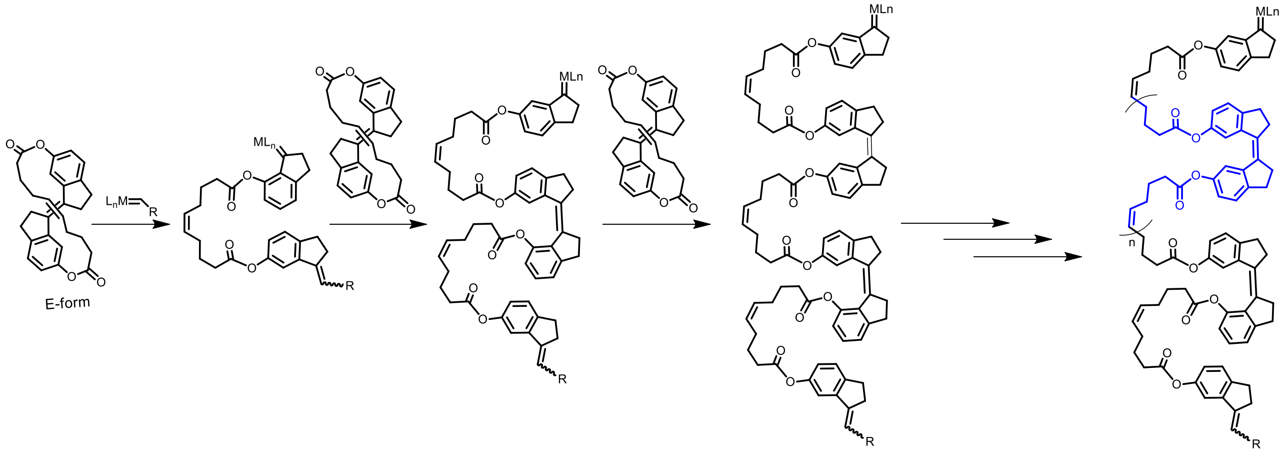

In both cases, the repeating unit is the same except the configurations at the double bonds.

8. ^{1}H NMR comparison of reactions of Z- and E-forms with Grubbs catalyst

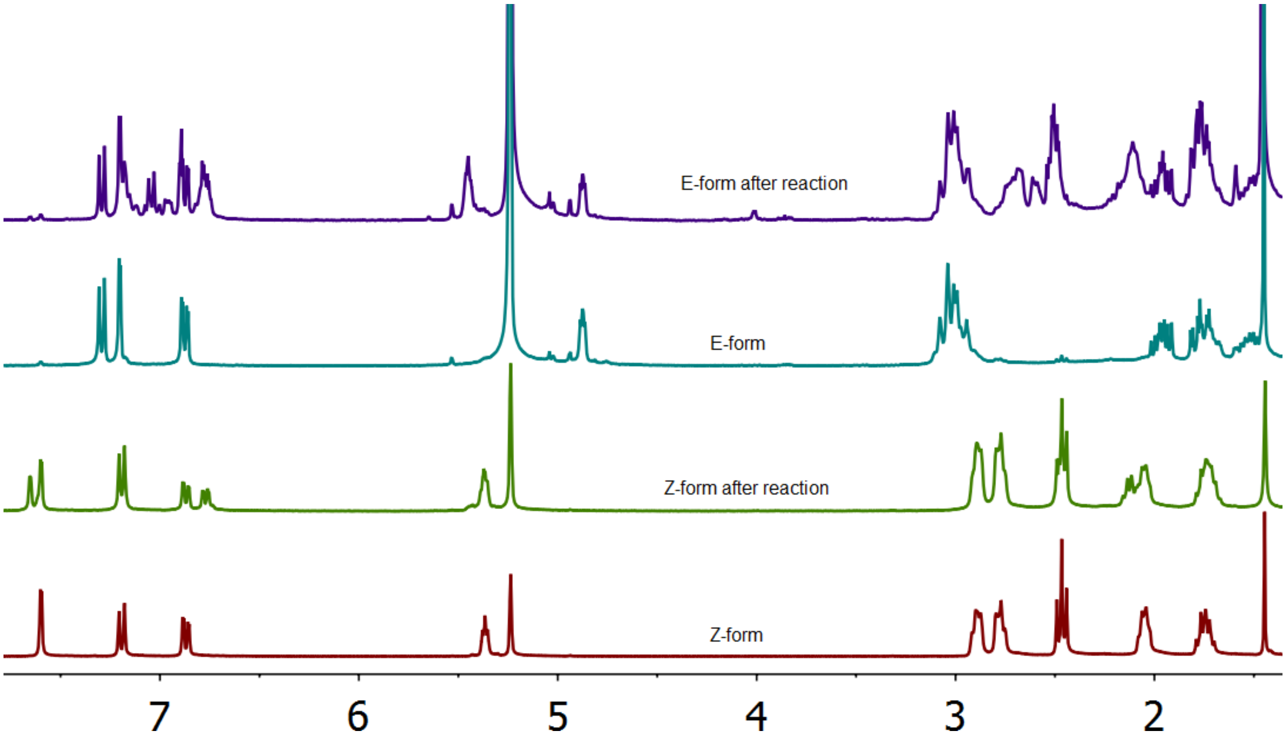

Figure S4. Comparison ^{1}H NMR spectra before and after reaction of E- and Z-forms. The reaction time was 15 minutes in both cases.

9. ESI-MS spectra of Z-form before and after the reaction

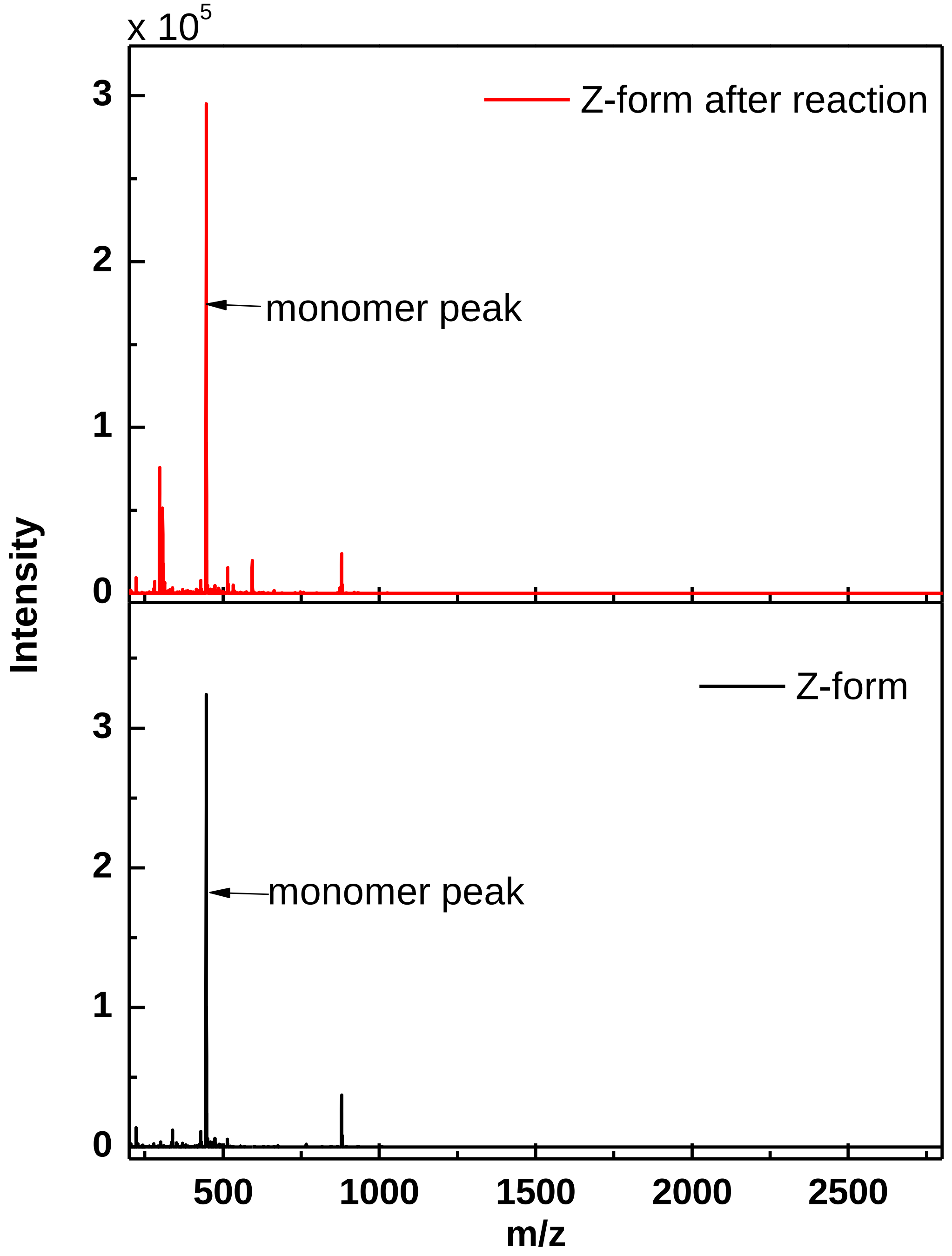

Figure S5. Comparison ESI-MS spectra of Z-form before and after the reaction. The reaction time is 15 minutes.

10. Spectra

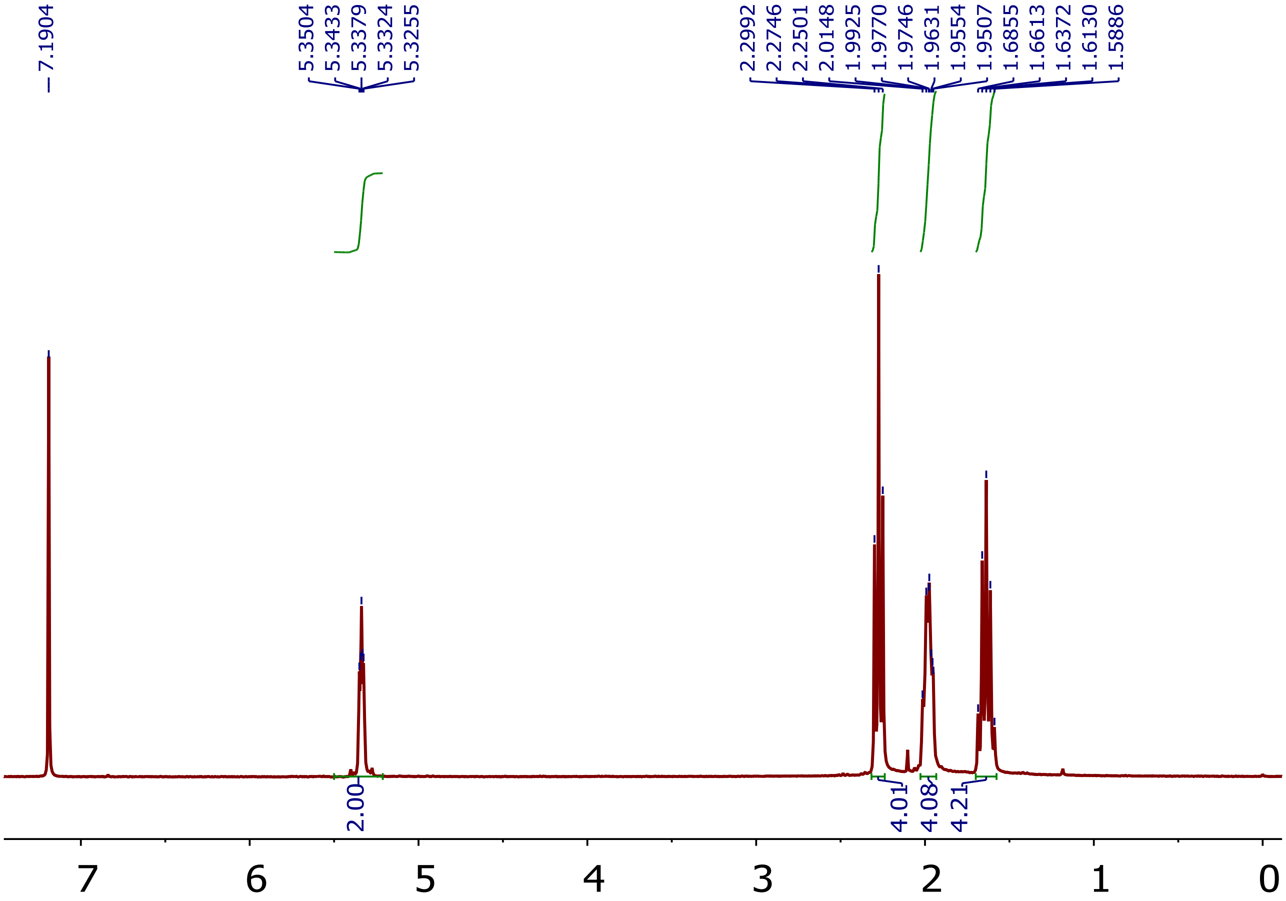

Figure S6. ^{1}H NMR spectrum of compound **3**.

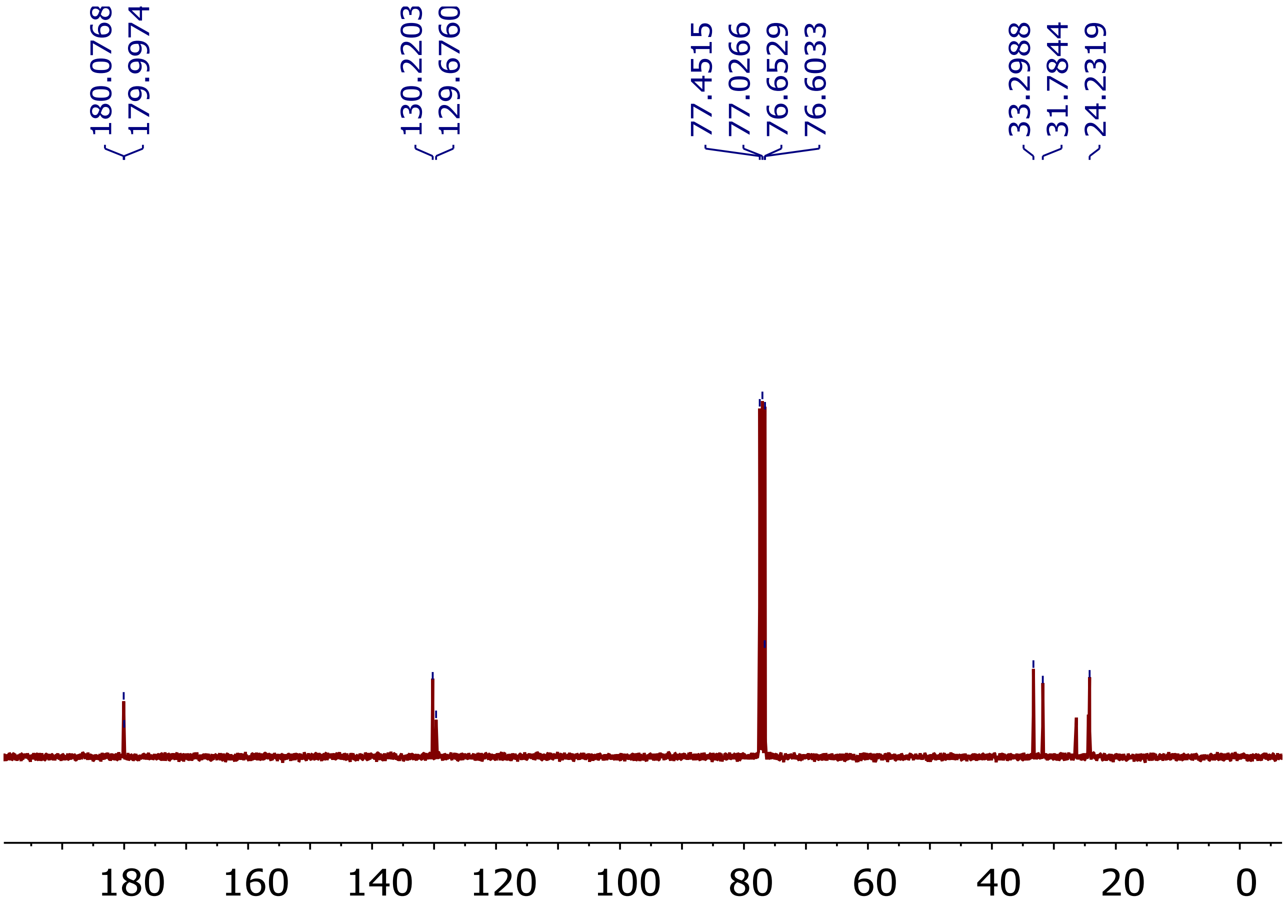

Figure S7. ^{13}C NMR spectrum of compound **3**.

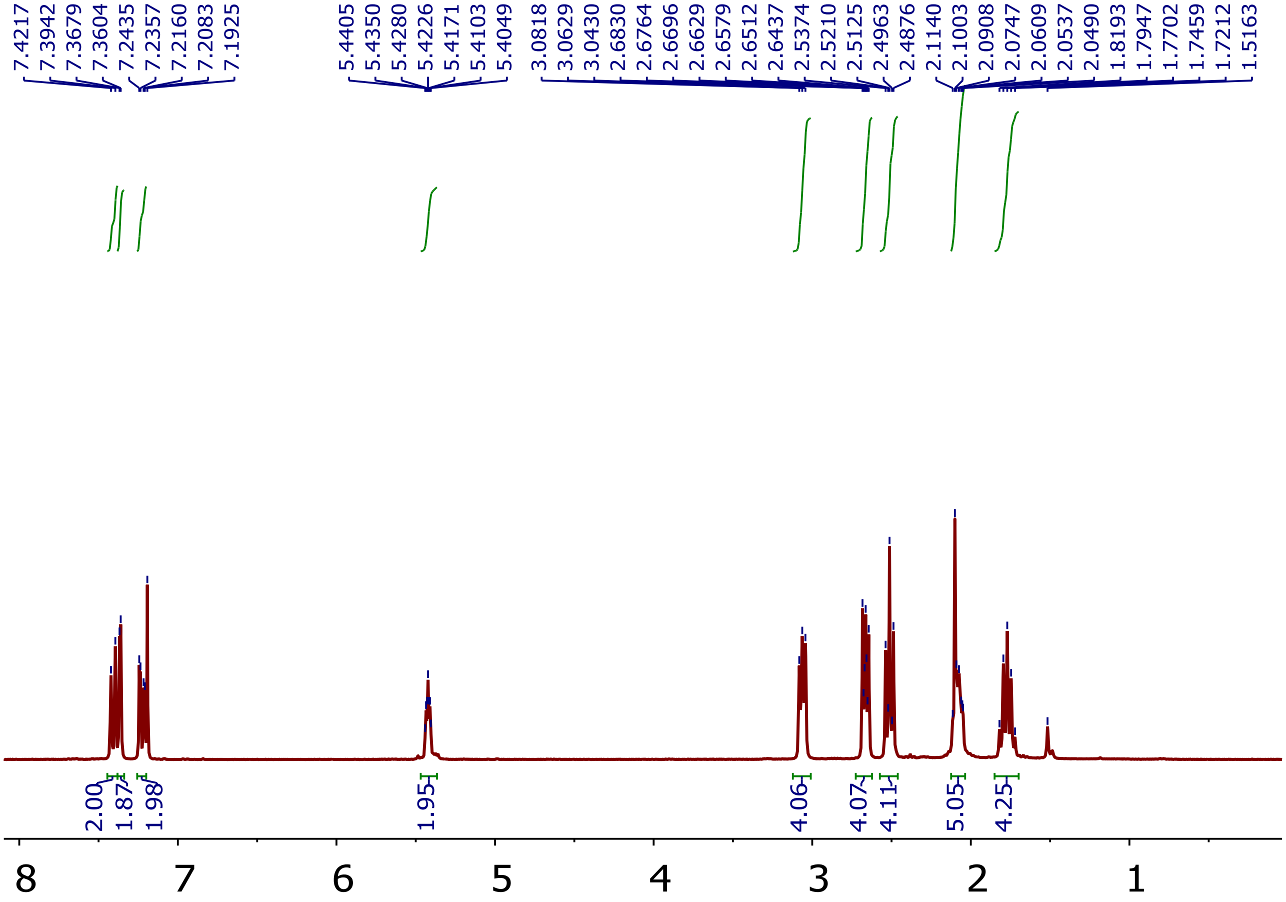

Figure S8. ^{1}H NMR spectrum of compound **4**.

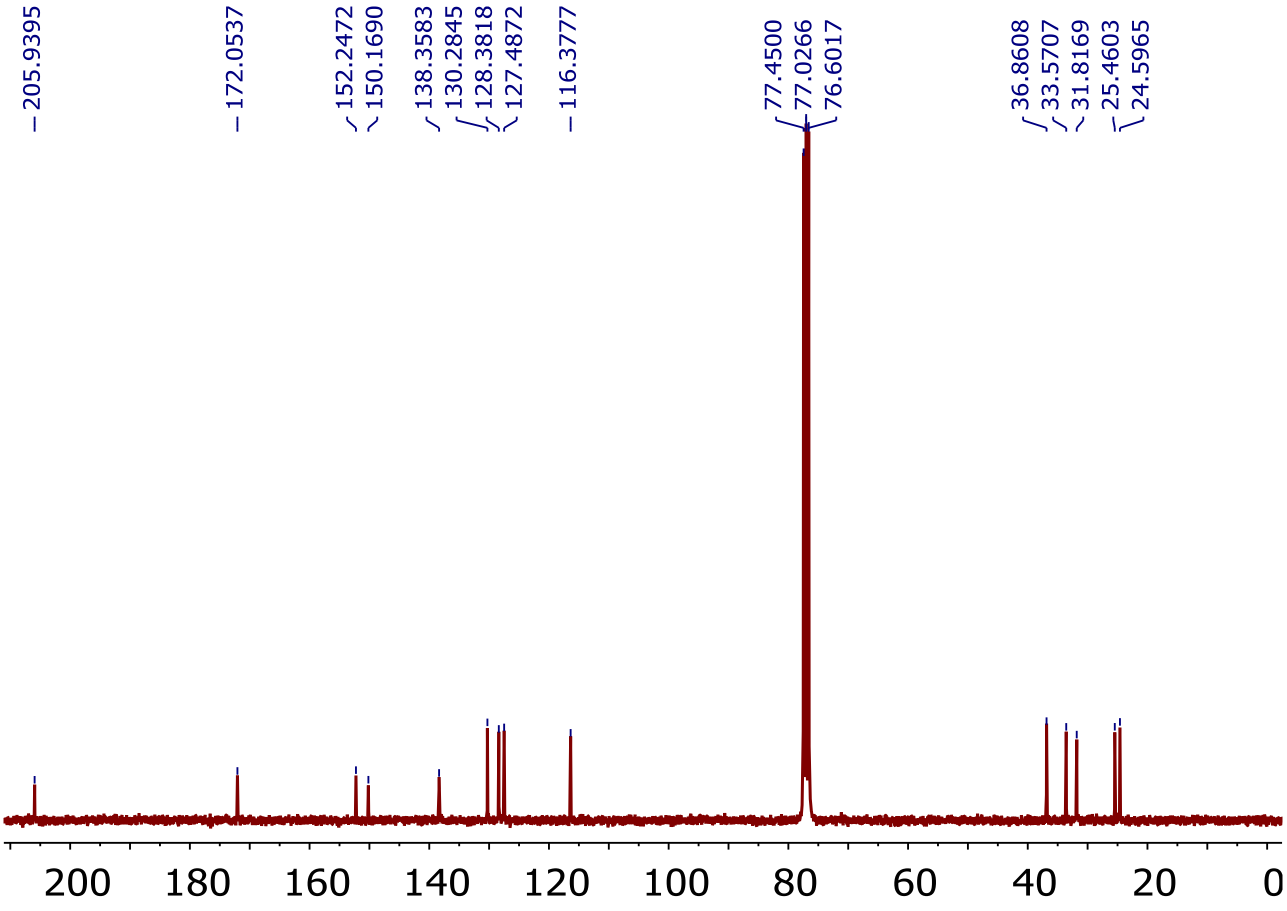

Figure S9. ^{13}C NMR spectrum of compound **4**.

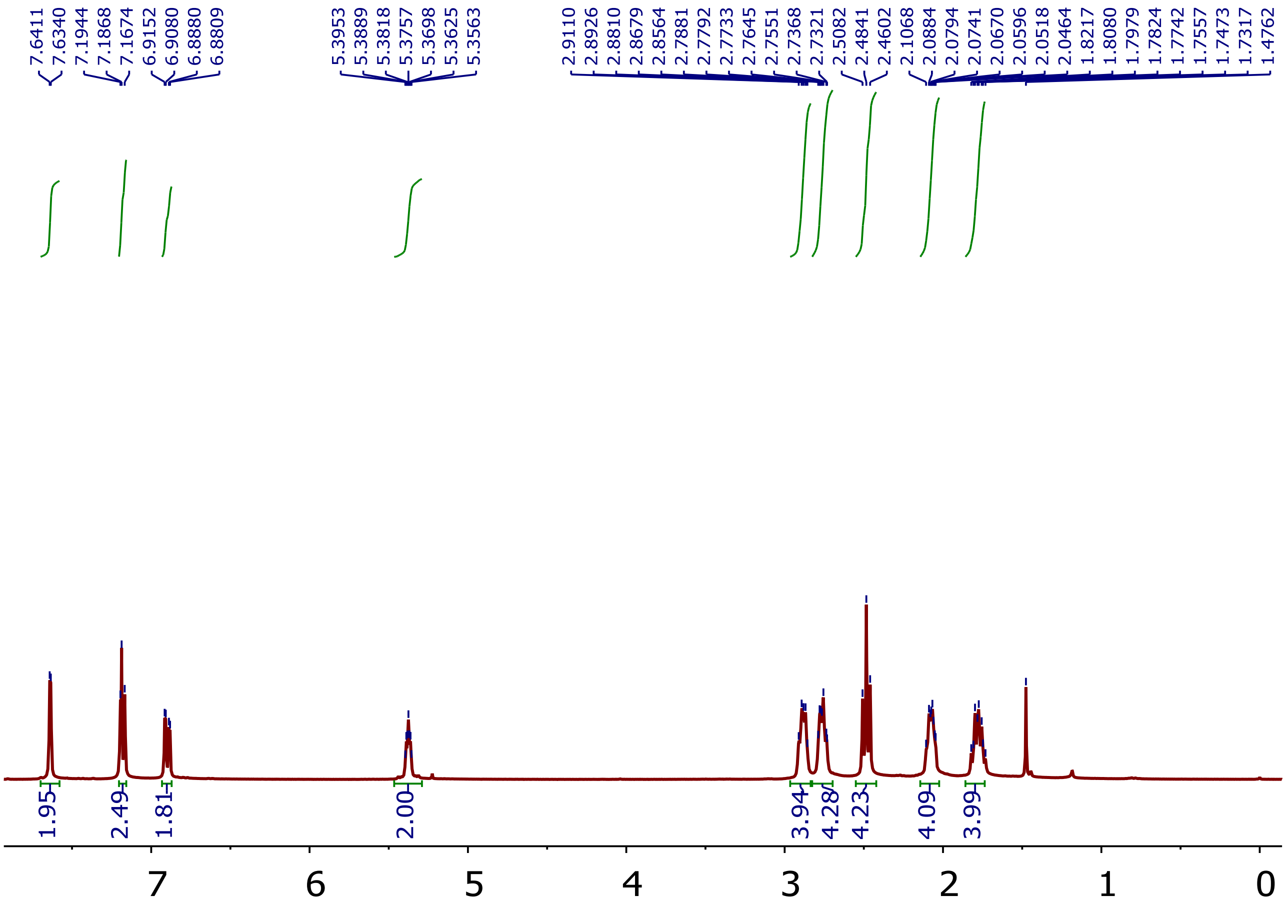

Figure S10. ^{1}H NMR spectrum of macrocycle **1** (Z-form).

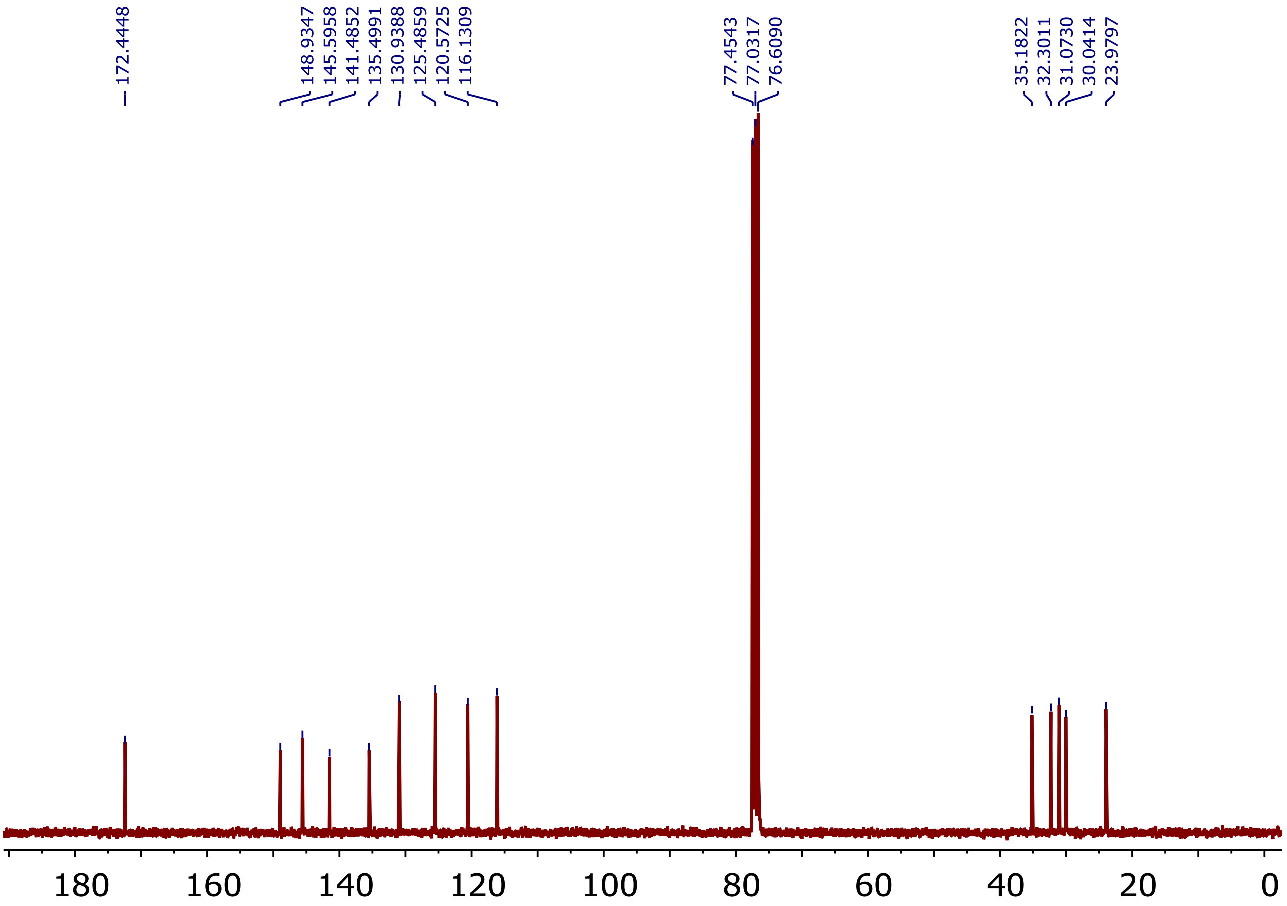

Figure S11. ^{13}C NMR spectrum of macrocycle **1** (Z-form).

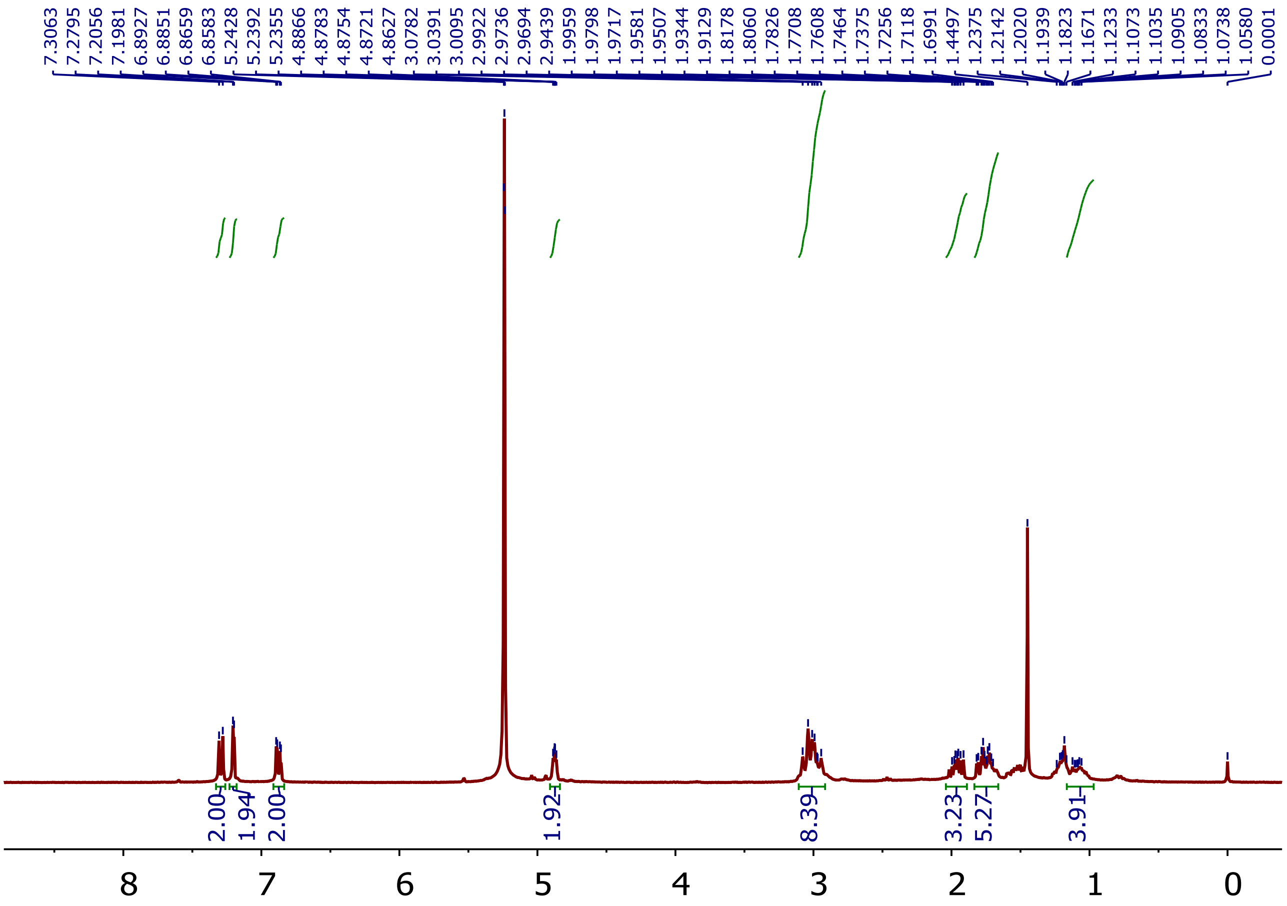

Figure S12. ^{1}H NMR spectrum of E-form.

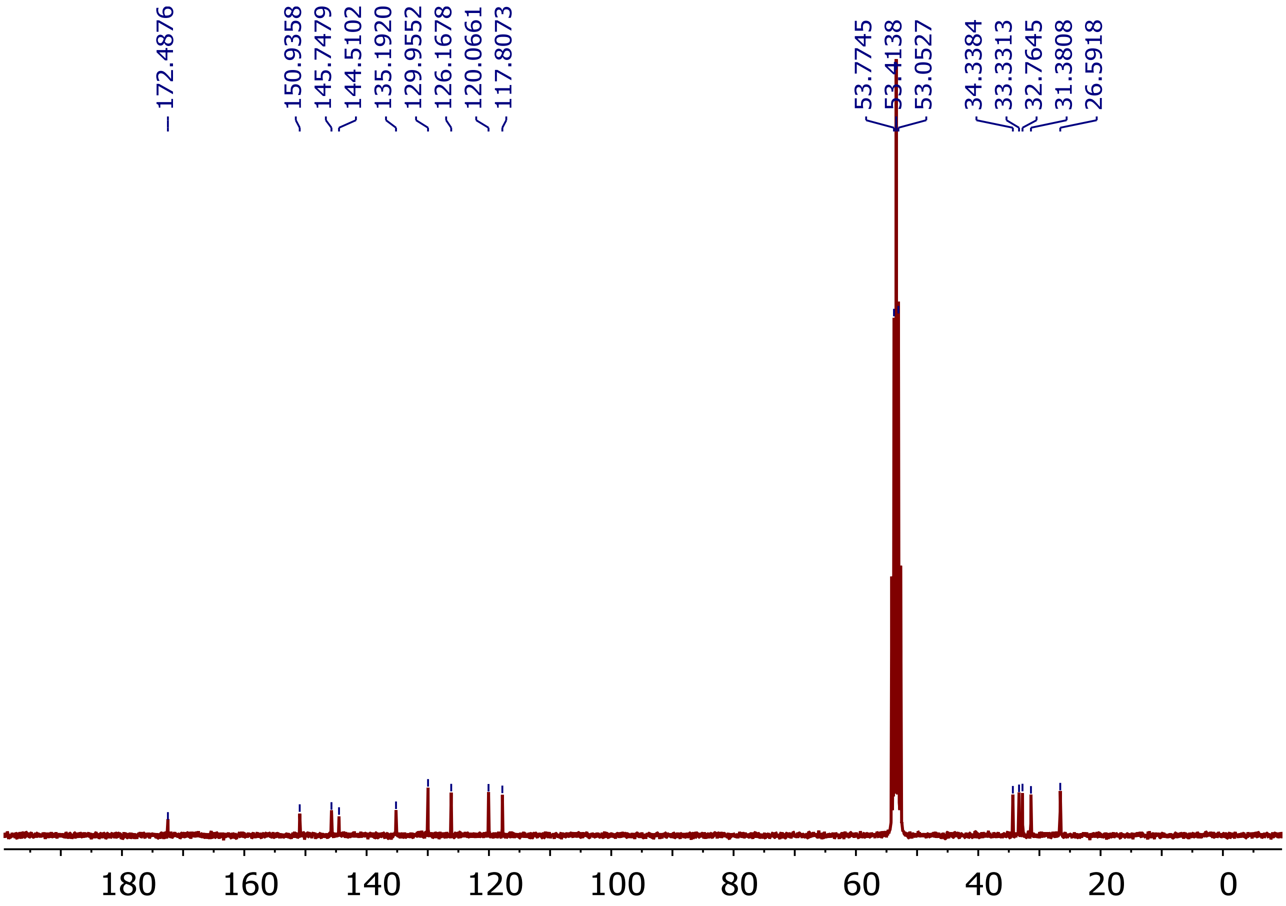

Figure S13. ^{13}C NMR spectrum of E-form.

11. References

[1] Gaussian 09, Revision A.02, M. J. Frisch, G. W. Trucks, H. B. Schlegel, G. E. Scuseria, M. A. Robb, J. R. Cheeseman, G. Scalmani, V. Barone, G. A. Petersson, H. Nakatsuji, X. Li, M. Caricato, A. Marenich, J. Bloino, B. G. Janesko, R. Gomperts, B. Mennucci, H. P. Hratchian, J. V. Ortiz, A. F. Izmaylov, J. L. Sonnenberg, D. Williams-Young, F. Ding, F. Lipparini, F. Egidi, J. Goings, B. Peng, A. Petrone, T. Henderson, D. Ranasinghe, V. G. Zakrzewski, J. Gao, N. Rega, G. Zheng, W. Liang, M. Hada, M. Ehara, K. Toyota, R. Fukuda, J. Hasegawa, M. Ishida, T. Nakajima, Y. Honda, O. Kitao, H. Nakai, T. Vreven, K. Throssell, J. A. Montgomery, Jr., J. E. Peralta, F. Ogliaro, M. Bearpark, J. J. Heyd, E. Brothers, K. N. Kudin, V. N. Staroverov, T. Keith, R. Kobayashi, J. Normand, K. Raghavachari, A. Rendell, J. C. Burant, S. S. Iyengar, J. Tomasi, M. Cossi, J. M. Millam, M. Klene, C. Adamo, R. Cammi, J. W. Ochterski, R. L. Martin, K. Morokuma, O. Farkas, J. B. Foresman, and D. J. Fox, Gaussian, Inc., Wallingford CT, 2016.

[2] P. R. A, *Phys. Rev. A* **1988**, *38*, 3098-3100.

[3] A. F. Rodrigues-Oliveira, F. W. M. Ribeiro, G. Cervi, T. C. Correra, *ACS Omega* **2018**, *3*, 9075-9085.

[4] C. Xu, X. Shen, A. H. Hoveyda, *J. Am. Chem. Soc.* **2017**, *139*, 10919-10928.
